# Intraventricular muscle bundle as a novel cause of left ventricular aneurysm

**DOI:** 10.1186/s12872-020-01678-9

**Published:** 2020-08-31

**Authors:** Junyu Pei, Xiaoyan Zheng, Yuting Liu, Daoquan Peng, Bilian Yu

**Affiliations:** 1 Department of Cardiovascular Medicine, The Second Xiangya Hospital, Central South University, 139 Middle Renmin Road, Changsha, 410011 Hu’nan China; 2 Department of Lymphoma and Hematology, Hu’nan Cancer Hospital, The Affiliated Cancer Hospital of Xiangya School of Medicine, Central South University, Changsha, China

**Keywords:** Left ventricular aneurysm, Abnormal left ventricular muscle bundle, Hemodynamics

## Abstract

**Background:**

There are a variety of causes of left ventricular aneurysm, but it is rarely due to a disturbance in intraventricular hemodynamics. To the best of our knowledge, there have been no reports of ventricular aneurysm at the left ventricular apex caused by an abnormal left ventricular muscle bundle.

**Case presentation:**

We report two cases of patients with congenital abnormal left ventricular muscle bundles which caused disturbances in intraventricular hemodynamics. This process eventually led to a left ventricular aneurysm at the apex of the heart. In both cases, transthoracic echocardiography (TTE) and cardiac magnetic resonance imaging (CMR) indicated ventricular aneurysm formation at the apex of the left ventricle. There were also abnormal muscular bundles connecting the ventricular septum and the posterior wall of the left ventricle. The only differences between these two cases were the comorbidities and severity of symptoms.

**Conclusion:**

Ventricular aneurysm at the apex of the left ventricle is common. However, it is rare for a ventricular aneurysm to form due to intraventricular hemodynamic disturbances caused by an abnormal muscle bundle as opposed to that due to original ventricular wall damage, which is more common. There is currently a lack of relevant studies on the treatment and prognosis of such patients. Whether surgical resection of a ventricular aneurysm leads to a better prognosis remains uncertain.

## Background

Currently, the common definition of ventricular aneurysm adopted by most clinicians is any large area of left ventricle (LV) akinesia or dyskinesia that reduces the LV ejection fraction. Myocardial infarction (MI) is the most common cause of ventricular aneurysm; other underlying diseases include sarcoidosis, trauma, left coronary artery originating from the pulmonary artery, Chagas disease, or myocarditis [1]. Idiopathic left ventricular aneurysms that have no clear underlying cause are rare [2]. In addition, apical ventricular aneurysms caused by hypertrophic cardiomyopathy have gained recent attention [3]. In this report, we introduce a previously undetermined potential cause of ventricular aneurysm.

## Case prestation

### Case 1

**Fig. 1:**
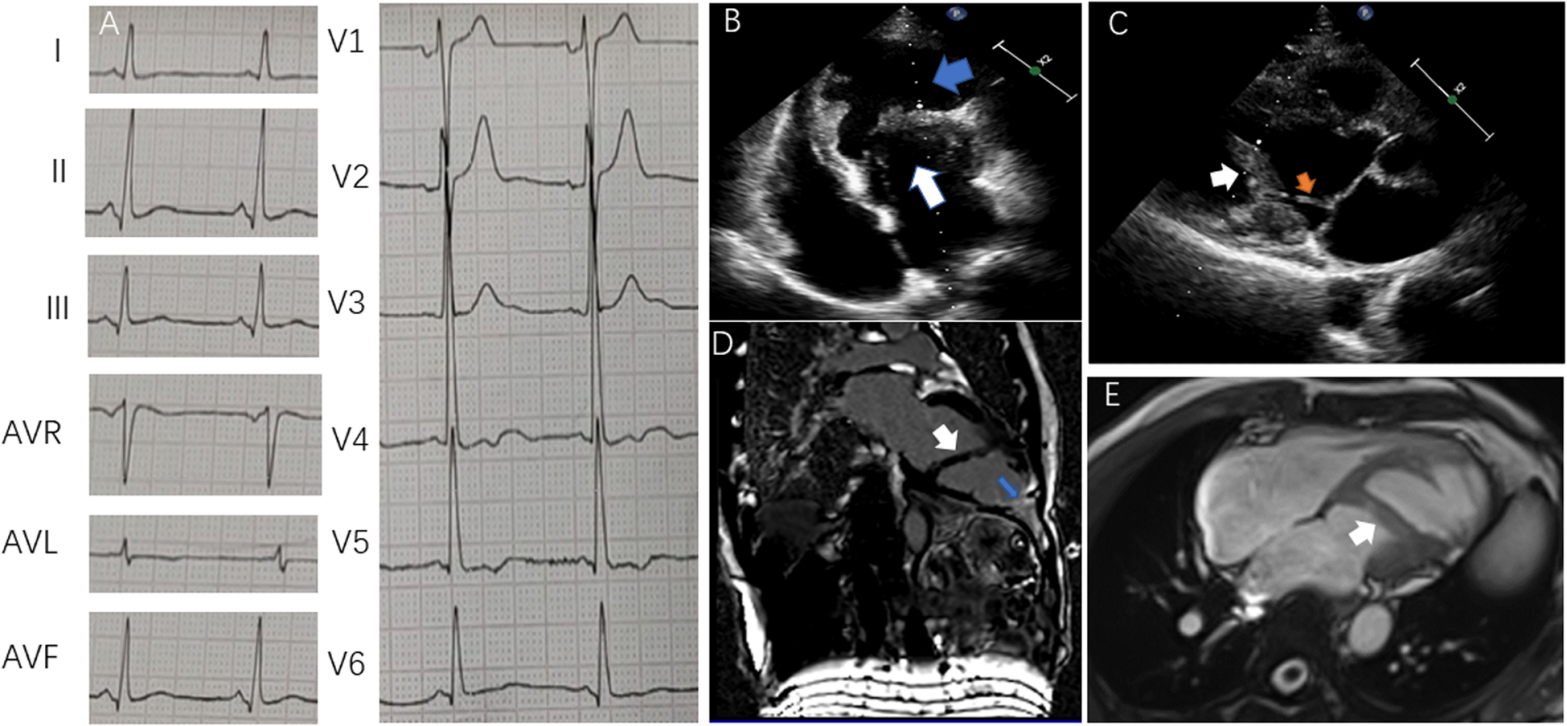
ECG, TTE and CMR of Case 1. **a**: ECG of case 1: Sinus rhythm, non-specific T wave abnormalities. **b**: TTE of case 1: Four-chamber view of TTE; the white arrow indicates the proximal chamber, while the blue arrow indicates the distal chamber. **c**: TTE of case 1: The yellow arrow shows the abnormal muscle bundle connected to the mitral valve. **d, e**: CMR of case 1: The white arrow indicates the abnormal muscle bundle, while the blue arrow indicates the transmural late myocardial enhancement. CMR, cardiac magnetic resonance imaging; ECG, electrocardiogram; TTE, transthoracic echocardiography

A 67-year-old man developed chest tightness and recurrent cerebral infarction. No obvious cause of cerebral infarction was found in the local hospital, so the patient was subsequently referred to our hospital for further evaluation. The patient had no family history of heart disease. A physical examination demonstrated left-downward displacement of the cardiac apex. Subsequently, transthoracic echocardiography (TTE) displayed a slightly enlarged left ventricle (left ventricular end-diastolic diameter was 55 mm) with decreased segmental motion of the ventricular wall and ventricular aneurysm formation at the apex of the left ventricle. Cardiac function was reduced, with a left ventricular ejection fraction (LVEF) of 45%. The left ventricle had several abnormal muscle bands connecting the posterior wall of the left ventricle and the interventricular septum, which separated the left ventricle into two parts. There was a relatively small communication between the main chamber and the aneurysm, with a diameter of 20 mm in diastole, and color Doppler flow imaging (CDFI) showed no obstruction to blood flow in the communication. Doppler detected a velocity of 190 cm/s during systole (Fig. 1).

To identify the cause of the ventricular aneurysm formation, we performed an electrocardiogram (ECG), coronary angiography (CAG) and cardiac magnetic resonance imaging (CMR) to identify a common etiology. There was no obvious abnormality in the ECG, and CAG showed no obstructive lesions. Even after an extensive investigation, we could not identify any common cause of ventricular aneurysm formation. We repeatedly inquired about the medical history of the patient. He complained of discomfort in the precordium since he was young and underwent TTE in 1989 and 1995. The TTE indicated the existence of a large abnormal muscle bundle that separated the left ventricle into two lumens including a proximal chamber and a distal chamber; however, there was no ventricular aneurysm at that time. The diameter of the communication measured 15 mm in diastole. Different from the present situation, we detected a mean systolic gradient of 35 mmHg and a diastolic gradient of 10 mmHg between the two chambers by continuous wave Doppler. Therefore, we believe that the formation of the apical ventricular aneurysm was related to hemodynamic changes caused by the multiple abnormal bundles in the left ventricle. Subsequent CMR confirmed the existence of the abnormal muscle bundles and the apical ventricular aneurysm with a wall thickness of 4 mm (Fig. 1). During diastole, blood flowed into the distal chamber by passing a bottle neck from the proximal chamber, and during systole, blood was squeezed from the distal chamber into the proximal chamber. Due to the blockage by the abnormal muscle bundles, the distal chamber needed enhanced contraction to push blood into the main chamber during systole, which resulted in a chronic increase in contractive pressure in the distal chamber and led to the formation of the apical ventricular aneurysm. This process is akin to the formation of an apical aneurysm in hypertrophic cardiomyopathy with mid-ventricular obstruction (MVO). We believe that the patient’s multiple cerebral infarctions were also associated with hemodynamic changes in the distal chamber. The stagnation of blood may have led to the formation of small thrombi in the aneurysm. Open surgery for the correction of the aneurysm was not proposed because cardiac function was stable. Instead, warfarin was prescribed to prevent intraventricular thrombus formation, with subsequent long-term follow-up.

### Case 2

**Fig. 2:**
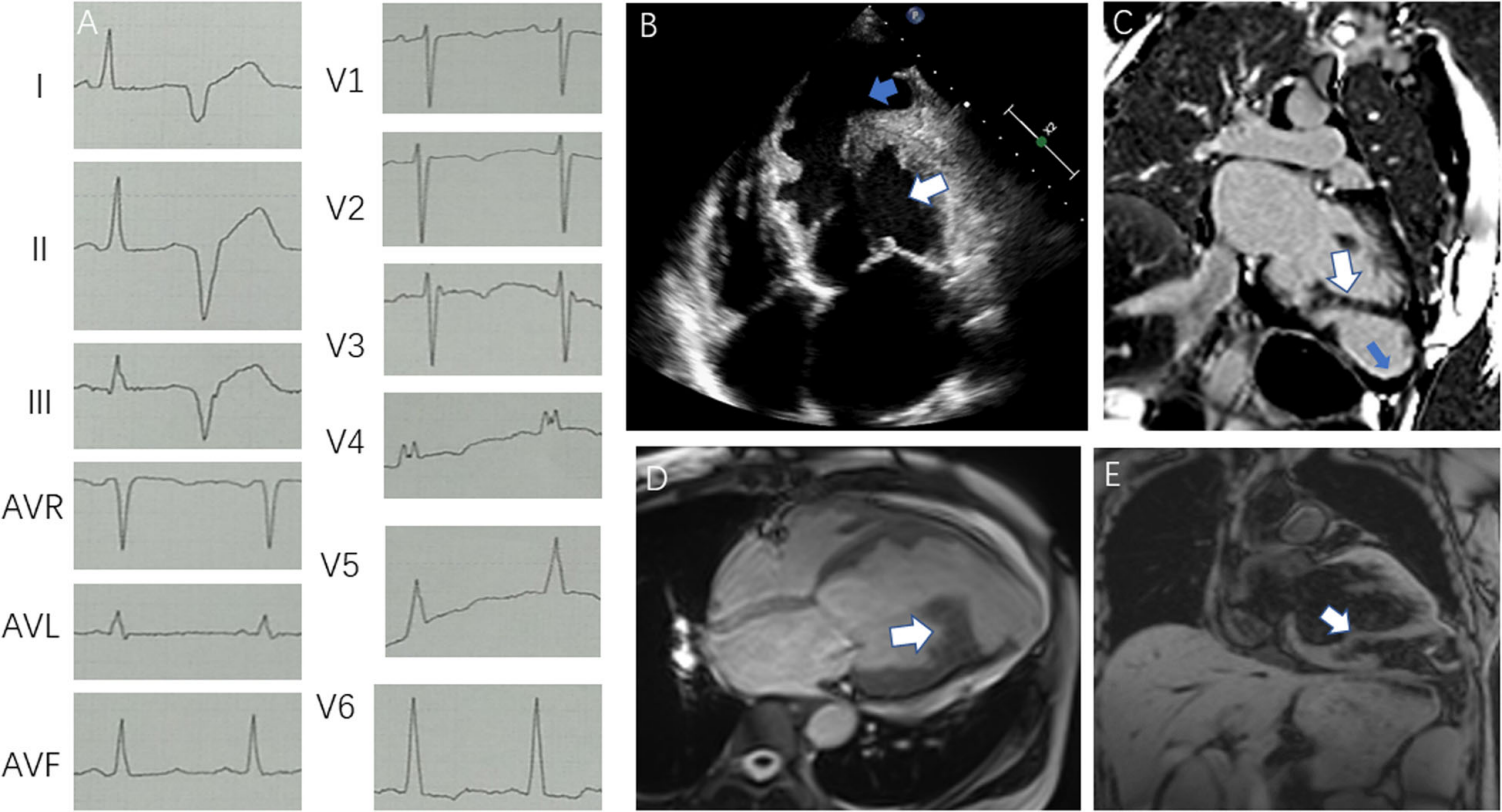
ECG, TTE and CMR of Case 2. **a**: ECG of case 2: Sinus rhythm, premature ventricular beats, poor V1-V3 R wave progression. **b**: TTE of case 2: Four-chamber view of TTE; the white arrow indicates the proximal chamber, while the blue arrow indicates the distal chamber. **c, d, e**: CMR of case 2: The white arrow shows the abnormal muscle bundle, while the blue arrow shows the transmural late myocardial enhancement. CMR, cardiac magnetic resonance imaging; ECG, electrocardiogram; TTE, transthoracic echocardiography

A 47-year-old woman suffering from recurrent shortness of breath over a period of 2 months was admitted to our hospital. Initially, she was diagnosed with coronary heart disease (CHD) at a local hospital and received standard medical therapy for CHD (including aspirin, atorvastatin, ACE inhibitor and vasodilators). However, subsequent coronary angiography showed no significant stenosis of the coronary arteries. Because coronary spasm and microvascular lesions could not be ruled out, doctors continued to give her routine treatment for CHD. The patient’s symptoms persisted after treatment at the local hospital, and she was subsequently referred to our hospital for treatment. TTE showed a left ventricle diastolic diameter of 64 mm and diffuse hypokinesis, with an LVEF of 34%. It is noteworthy that an apical ventricular aneurysm with a wall thickness of 3 mm had formed. Multiple, developed muscle bundles were found in the left ventricle, which also divided the left ventricle into two distinct chambers. Blood entered the apical aneurysm in diastole and was ejected out of it in systole, as it occurs in the normal LV. The diameter of the communication measured 25 mm in diastole. The flow rate from the main chamber to the apical aneurysm was 150 cm/s. The patient also had no family history of heart disease. Subsequent CMR suggested that the myocardial three-layer structure in the left ventricular apex disappeared with ventricular aneurysm formation (Fig. 2), which was strikingly similar to case 1. We conducted investigations to find the common causes of ventricular aneurysm, but there were no special findings other than moderate anemia (Hemoglobin: 8 g/dL, normal range: 11–15 g/dL) due to thalassemia. The reason for the more obvious decrease in cardiac function in this patient may partially be related to anemia, which would increase cardiac output and is one of the causes of cardiomyopathy [4]. Although there was no previous echocardiogram showing the presence of the intraventricular muscle bundles, we assumed that case 2 shared a similar underlying mechanism with case 1 because her imaging findings were almost identical to his. The only differences from case 1 were the symptoms and treatment strategy for this patient. Since this patient was younger and had more severe symptoms, we suggested an open surgery to dissect the intraventricular bundle and aneurysm but the patient declined. The symptoms of heart failure have improved significantly with treatment during the 3 months of follow up.

## Discussion and conclusion

The formation of ventricular aneurysms in these two patients was associated with hemodynamic changes, a mechanism completely different from ventricular aneurysms caused by anterior myocardial infarction. The impact of abnormal muscle bundles on left ventricular hemodynamics in these patients is very similar to that seen in MVO in patients with hypertrophic cardiomyopathy. We speculate that the left ventricular apical aneurysm and associated regional myocardial scarring developed secondary to increased apical chamber ventricular wall stress as a result of the elevated intracavitary systolic pressure from blockage by the abnormal muscle bundles. The increased wall stress imposed an increased pressure load on the apical myocardium, resulting in increased oxygen demand and impaired endocardial blood supply. The chronic damage to the myocardium led to aneurysm formation [5]. Although the hemodynamic changes are very similar to those of MVO, there are obvious differences in clinical symptoms and structure morphology between these two diseases. For example, MVO usually presents with hypertrophy of left ventricular wall and symmetric aneurysm, while an intraventricular muscle bundle presents with normal left ventricle thickness and asymmetric aneurysm.

There have been a few reported cases of double-chamber left ventricles similar to our two cases [6]. Previous case reports suggested that this situation was more common among young patients, but there are no corresponding reports on the long-term prognosis of this disease [7]. As shown in case 1, there was a significant pressure gradient between the two chambers of the left ventricle when he was young. As time went by, the apical ventricular aneurysm gradually formed. As a result of ventricular dysfunction and increased size of the communication due to ventricular enlargement, the pressure gradient between the two chambers disappeared. Based on the natural history of Case 1, we strongly suspect that double-chamber left ventricle may manifest as left ventricular aneurysm in later stages. However, not all patients with double-chamber left ventricle will develop ventricular aneurysm. The time of occurrence and degree of the clinical symptoms might be associated with the size of the communication and the pressure gradient between two chambers [8]. More follow-up studies are needed for the prognosis and outcome of double-chamber left ventricle.

**Fig. 3:**
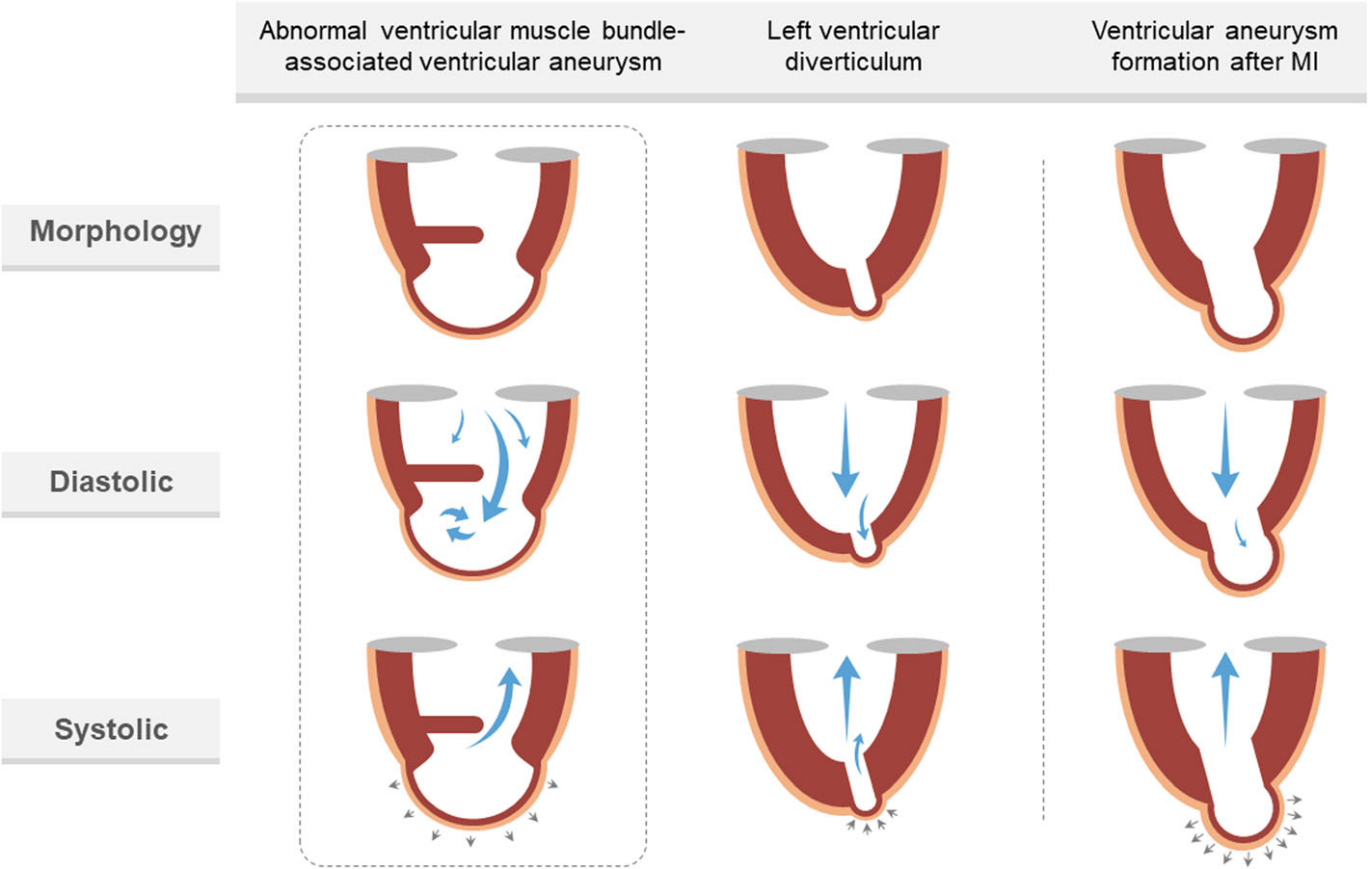
Morphological and hemodynamic changes in abnormal ventricular muscle bundle-associated ventricular aneurysm, left ventricular diverticulum and ventricular aneurysm formation after MI. In both abnormal muscle bundle-associated ventricular aneurysm and left ventricular diverticulum, blood flows from the main chamber into the sub-chamber during the systolic phase, and high-speed blood flow can be detected in the communication. The direction of diastolic blood flow is opposite. However, blood flow into and out of the post-MI aneurysm is not obvious. MI, myocardial infarction

It is relatively straightforward to identify a ventricular aneurysm caused by MI or an abnormal muscle bundle. Patients with post-MI aneurysm usually have a history of chest pain, characteristic ECG changes, and more importantly, the presence of an obstructed coronary artery. Meanwhile, a patient with an intraventricular muscle bundle-related aneurysm usually has no history of chest pain, no pathologic Q wave in precordial leads, and normal coronary arteries. Although the myocardial portions of both types of aneurysm show significant thinning, post-MI aneurysms are characterized by localized akinesia or dyskinesia (outward movement) during systole [9]. In addition to the differentiation from post-MI aneurysm, left ventricular diverticulum should be considered. A diverticulum is a rare cardiac malformation defined by a sac-like out-pouching with normal contractility arising from the wall of the left ventricle. Unlike abnormal muscle bundle-associated ventricular aneurysm, all three layers of the heart are present in the myocardial portion of a diverticulum [8]. Meanwhile, the diverticulum is always located in the apical muscular and posterior-inferior wall, which is not separated by an abnormal structure. Table 1 lists the key points for differentiation of these left ventricular dysmorphologies. Morphological and hemodynamic changes in these three diseases are shown in Fig. 3.

**Table 1:** Differentiation points for left ventricular dysmorphologies by TTE and ECG

	Abnormal ventricular muscle bundle-associated ventricular aneurysm	Left ventricular diverticulum [10]	Ventricular aneurysm formation after MI [11]
Sub-chamber position	Apex or side wall	Apical or lower posterior wall, outward bulging	Apex
Wall thickness	Thin, myocardial three-layer structure disappears	Normal or thin; myocardial three-layer structure exists	Thin, myocardial three-layer structure disappears
Wall motion	Normal or reduced	Normal	Absent or outward movement
Communication between two chambers	Big or small	Small	Big
Echo of Muscular trabeculae	Normal or slightly increased	Normal	Normal
ECG	No Q wave in precordial leads	No Q wave in precordial leads	Q wave in precordial leads

Furthermore, the symptoms of cardiac insufficiency and systemic emboli in these two patients remind us of the need to consider non-compact cardiomyopathy (NCM), which is of genetic origin. The characteristic features of NCM include a two-layered ventricular wall, comprising a thinner compact epicardial layer and an inner non-compact layer, with prominent trabeculations associated with deep, inter-trabecular recesses. Echocardiography and CMR are effective tools for detection of these morphologic diagnostic criteria [12].

There are currently no reports on treatment for this kind of abnormality. Resection of the abnormal muscle bundle might be helpful to prevent aneurysm formation, but open surgery seems impractical before the aneurysm forms unless severe obstruction exists. Resection of the aneurysm is a reasonable approach for those with symptomatic heart failure or arrythmia. Since the first patient had only mild symptoms of heart failure and exhibited less myocardial necrosis on CMR, we recommended an anticoagulant drug and ACE inhibitor, as well as scheduled follow-up. However, the second case was a younger patient with more severe symptoms of heart failure. Therefore, we recommended surgical resection of the aneurysm, but she declined, as she felt much better after 1 month of medical treatment. Both patients have been discharged and have remained in good condition for the past few months.

In summary, given that the morbidity of abnormal ventricular muscle bundle-associated ventricular aneurysm is extremely rare, clear data about the prognosis, risk factors, and treatment of this condition are unavailable. The effectiveness of drugs that attenuate ventricular remodeling needs further research. Surgical treatment, however, is indicated when other cardiovascular abnormalities or left cardiac insufficiency is present. The timing of operation is decided based on the cardiac function of the patient, the risk of ventricular aneurysm rupture and the degree of obstruction between the two chambers. The patient’s need for anticoagulation therapy depends on his presentation. Anticoagulation may be necessary in the event of repeated embolization.

## Data Availability

Not applicable.

## Abbreviations

TTE: Transthoracic echocardiography
CMR: Cardiac magnetic resonance
LV: Left ventricle
MI: Myocardial infarction
LVEF: Left ventricular ejection fraction
CDFI: Colour Doppler flow imaging
ECG: Electrocardiogram
CAG: Coronary angiography
LGE: Late gadolinium enhancement
MVO: Mid-ventricular obstruction
